# Infection or Malignancy? Malignant Pulmonary Mass Mimicking Pneumonia

**DOI:** 10.1055/s-0036-1572359

**Published:** 2016-02-03

**Authors:** Serdar Evman, Korkut Bostanci, Mustafa Yuksel

**Affiliations:** 1 Department of Thoracic Surgery, Sureyyapasa Training and Research Hospital, Istanbul, Turkey; 2 Department of Thoracic Surgery, Marmara University School of Medicine, Istanbul, Turkey

**Keywords:** exploratory thoracotomy, lung cancer, positron emission tomography, pulmonary cavity

## Abstract

A 36-year-old woman, unresponsive to pneumonia antibiotherapy followed by antituberculosis treatment, was referred to our clinic. Thorax computed tomography (CT) and positron emission tomography CT showed cystic mass and mediastinal lymph node with suspicion of malignancy. Fine needle aspiration biopsy and mediastinoscopy showed no malignancy, so the patient underwent an exploratory thoracotomy. A frozen section of wedge-resected mass was reported as adenocarcinoma, leading to right lower lobectomy with mediastinal lymph node dissection. Besides cutting-edge diagnostic techniques, exploratory thoracotomy for cavitary lung lesions can still be necessary, as the last-line choice. The probability of malignancy must always be considered, despite a patient's age or symptoms.

Lung infections can easily mimic malignancies, but malignancies mimicking infections are relatively uncommon. 1 Different lung diseases have radiologic signs and symptoms simulating lung cancer, making diagnosis difficult. Tissue sampling is necessary to diagnose cavitary lesions and to choose the right treatment. 2 Sputum and bronchoalveolar lavage cultures do not always give correct results, thus needle biopsy or even video-assisted thoracoscopic surgery (VATS) is often necessary. This report presents a case of cavitary lung cancer prediagnosed as pneumonia and tuberculosis; the final diagnosis could only be made with exploratory thoracotomy.

## Case Report

**Fig. 1:**
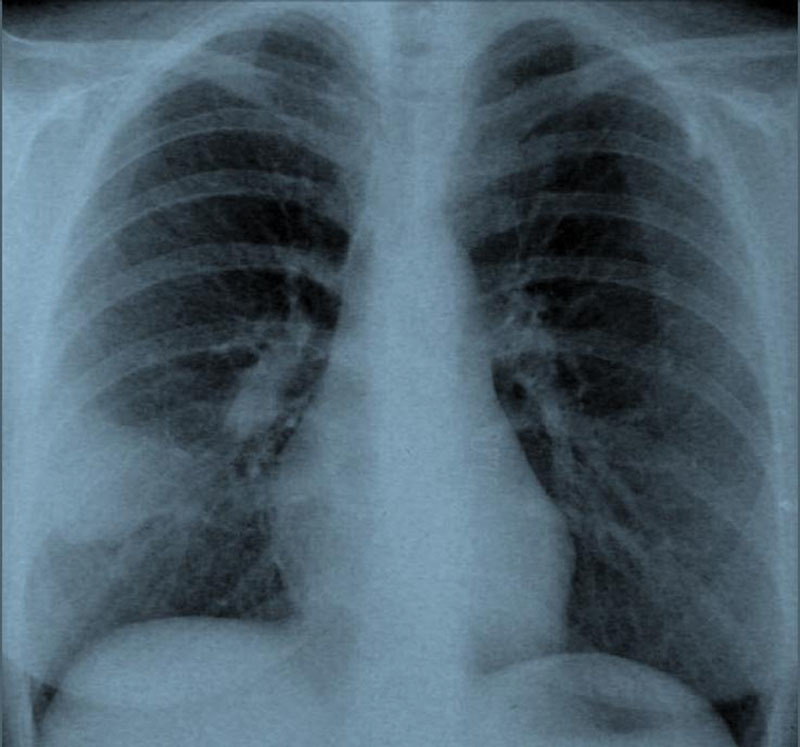
Chest X-ray of the patient at admission to the pulmonology clinic.

A 36-year-old nonsmoking woman was admitted to a pulmonology clinic with high fever, chest pain, and productive cough. Her white blood count was 9,000/μL, C-reactive protein was 2.9 mg/dL, and erythrocyte sedimentation rate was 58 mm/h. Oral antibiotic therapy was started because a chest X-ray revealed an opacity resembling pneumonia in the right lower zone ( Fig. 1 ).

**Fig. 2:**
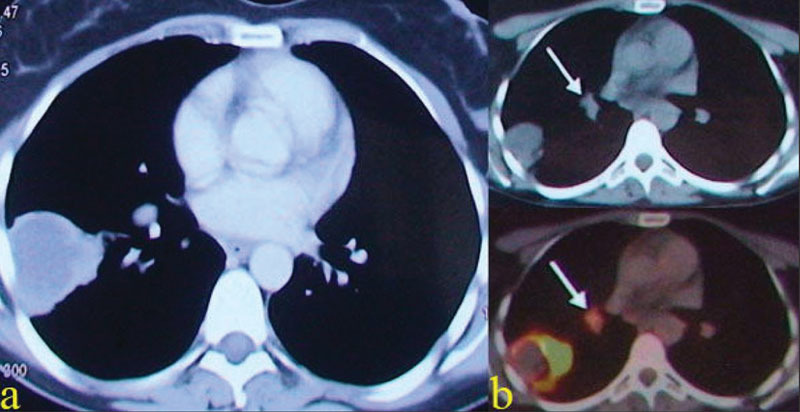
(a) Thorax computed tomography (CT) after antibiotic therapy and (b) positron emission tomography/CT after antibiotic and antituberculosis therapy showing the mass and hilar (no. 10) lymph node (white arrow).

However, chest X-rays and symptoms remained unchanged after 3 weeks of antibiotic therapy. A cavitary mass of 6.5 × 5.5 × 4 cm suspicious for malignancy was detected in the superior segment of the right lower lobe on thoracic computed tomography (CT) ( Fig. 2a ). The case was discussed at the thoracic tumor board, and a malignancy was highly suspected. Positron emission tomography (PET) CT scan revealed a cavitary mass in the right lower lobe with a high maximum standard uptake value (SUVmax: 22.7), consistent with malignancy ( Fig. 2b ). In addition, the right paratracheal and hilar (4R and 10R) lymph nodes also had high uptake. A bronchoscopic examination was planned, but the patient refused. A transthoracic fine needle aspiration biopsy was performed, which showed only reactive mesothelial cells, neutrophils, lymphocytes, and erythrocytes and no atypical cells. Because the fine needle aspiration biopsy was negative for malignancy, empirical multidrug antituberculosis therapy was initiated due to endemic tuberculosis in Turkey, but the patient stopped taking her medicine on day 25 of therapy due to gastrointestinal side effects.

The repeated thorax CT after antituberculosis therapy was still suspicious for malignancy, thus resection of the mass following a mediastinoscopy was suggested. Mediastinoscopy of nodes 2R, 4R, 4L, and 7 showed anthracosis and chronic fibrosis but no sign of malignancy. With exploratory thoracotomy, a wedge resection of the mass was performed and frozen section examination reported adenocarcinoma of the lung. The resection included a right lower lobectomy, with systematic lymph node dissection. Histopathologic examination revealed a T2N0 adenocarcinoma, and the patient was discharged on postoperative day 4 without any complications.

## Discussion

Radiologic findings suggesting lung cancer include a parenchymal mass showing spiculations, microlobulations, a thick-walled cavity, a cavity showing nodular margins, and chest wall invasion. 1 However, all these features are not specific to lung cancer, and nonmalignant diseases like infections, pulmonary infarctions, and abscesses may cause similar radiologic findings. 2 3 The differential diagnosis should be made according to radiologic findings and presentation of signs and symptoms.

In this report, we presented a case in which the patient had lost precious time with multiple incorrect diagnoses and treatments. The patient most probably had post–obstructive pneumonia at the beginning, and the antibiotics depressed but did not cure the symptoms. In a young, nonsmoking patient with productive cough and high fever as the only symptoms, bacterial pneumonia can be initially suspected. But after the chest X-ray showed such dense and large opacity, a thorax CT should have been ordered. Because this case was not responsive to antibiotics and biopsies showed no sign of malignancy, empirical treatment for pulmonary tuberculosis was initiated by the pulmonologists, which is still a common approach in endemic areas, as well as in Turkey. 4 More than 4 months were lost since the patient's symptoms began. Delay in treatment is a concern raised by various authors. 5 This delay could have been prevented by performing an earlier open lung biopsy followed by the necessary surgical resection.

Pulmonary tuberculosis is a major health problem with a rising incidence in developing countries throughout the world. Even though PET/CT revealed a high fluoro-2-deoxy- d -glucose uptake in our case, it was not specific for malignancy and it could have indicated an infection. Granulomatous diseases or tuberculoma, a fairly discrete nodule or mass with central caseous necrosis, is a well-known disease that shows intense fluoro-2-deoxy- d -glucose uptake leading to false-positive results. 6 7 8 PET/CT revealed true positivity for the mass and false positivity for the mediastinal lymph nodes in our case, probably due to previous infections and chronic lymphadenitis, demonstrating the importance of histopathologic examination for every PET-positive lesion.

Needle biopsies and cultures do not always provide accurate results. Even with results positive for infection, the probability of malignancy should not be ignored in cavitary lung lesions. In this case, percutaneous needle biopsy was not effective, most likely due to aspirating the central necrosis material without any vital malignant cells, and thus gave a misleading result. A minimally invasive approach (VATS) was not used because dense adhesions due to tuberculosis or an acute pulmonary infection was suspected. An exploratory thoracotomy was preferred for the diagnosis, so that it would be possible to treat the disease at the same session.

Even with the present modern diagnostic techniques such as CT, PET/CT, bronchoscopy, or VATS, exploratory thoracotomy can still be an important modality in the diagnosis and treatment of cavitary lung lesions. The likelihood of malignancy must always be kept in mind, despite the patient's age or symptoms.
